# Remarks on the Ophthalmy, Psorophthalmy, and Purulent Eye. With Methods of Cure Considerably Different from Those Commonly Used; and Cases Annexed, in Proof of Their Utility

**Published:** 1781-02

**Authors:** 


V. *Remarks on the Ophthalmy, Pſorophthalmy, and Purulent Eye. With Methods of Cure conſiderably different from thoſe commonly uſed; and caſes annexed, in proof of their utility. By* James Ware, *Surgeon,* 8vo. London, 133 *pages,* 1780.

AFTER a brief deſcription of the eye and its appendages, the author proceeds to treat of the ophthalmy or inflammation of that part of

 the the tunica conjunctiva which covers the globe of the eye: this disease occurs in very different degrees; sometimes it occupies only a part of the globe, but in common it extends over the whole. It may be superficial, affecting the conjunctiva only, or so deep as to reach the sclerotica and internal coats. In general the conjunctiva is not much thickened, but sometimes its membranous appearance is entirely destroyed. Whatever the degree of inflammation may be, it will, in general, be found that light is offensive to the eye, and to avoid the pain which it occasions, the injurious method of binding compresses or plaisters tight over the eye, has been but too often practised, with a view to keep out the light, as well as to prevent the motion of the eye. Our author, instead of such applications, which by confining the tears add to the irritation, and by their pressure increase the obstruction in the minute vessels on which they act, recommends a pasteboard hood or bonnet to be worn at a convenient distance from the eye. It must not be supposed however that the access of light is the only cause of pain; the sufferings of the patient are sometimes continual and excessive, acute pains darting through the eye to the back part of the head; these sensations, which always indicate much danger danger of the lofs of fight, may be confidered as the effects of inflammation.

In enumerating the caufes of ophthalmy, Mr. Ware obferves that it is fometimes epidemical, affecting a whole neighbourhood at the fame time, as was the cafe in 1778, at Newbury, and at feveral of the camps.—It may be brought on by blows or wounds, by extraneous bodies lodged in the eye, or it may be the confequence of other difeafes, as the fmall-pox, meafles, fcrophula, or lues.

Speaking of the means of cure, he firft takes notice of bleeding; he prefers topical to general bleeding, and recommends the application of leeches in preference to arteriotomy, on account of the inconveniencies that fometimes attend the latter. The number of leeches applied fhould feldom be lefs than three, and it will be proper to confine their application as near to each other as poffible in the hollow of the temple; when placed on or very near the eye-lids they are liable to occafion them to fwell, and to increafe the irritation of the eye.

Our author fpeaks unfavourably of bleeding the eye itfelf, as it is apt to irritate. If the conjunctiva is fcraped with a brufh made of barley beards, after the manner of fome, fome of the fpiculæ

ſpiculæ may be left in the eye, and Mr. Ware ſuſpects this to have happened to one of his own patients; dividing the blood veſſels with a lancet or needle, he thinks will be requiſite only when a ſpeck on the cornea is ſupplied with one or more diſtinct blood veſſels.—He has experienced good effects from bliſters of the ſize of half a crown, applied on the temples, directly over the orifices made by the leeches; he likewiſe recommends an attention to regimen, and the uſe of gentle laxatives, but his chief dependence ſeems to be upon the topical application of opium. For this purpoſe he recommends the Tinctura Thebaica of the London Diſpenſatory. Two or three drops of this medicine are to be dropped into the eye once or twice a day. When firſt applied, it cauſes a ſharp pain, and a copious flow of tears, which continues a few minutes and gradually abates, after which we are told that a great and remarkable degree of eaſe generally ſucceeds. The author informs us that the inflammation is often viſibly abated by a ſingle application of this tincture, and that many bad caſes have been completely cured by it in a fortnight, after every other kind of remedy had been uſed for weeks and ſometimes months without any ſucceſs. This ſpeedy good effect, however, is not to be expected expected in all cases indiscriminately. In some the amendment is more slow and gradual, and a few instances have occurred, in which no relief at all was obtained from its first application. In cases of the latter kind, in which the complaint is generally recent, the eyes appear shining and glossy, and feel exquisite pain from the rays of light. However, notwithstanding these symptoms, the application is sometimes found to succeed; and whether it will or not, can only be determined by making the trial, which is attended with no other inconvenience than the momentary pain it occasions: and when it is found to produce no good effect, the use of it must be suspended, until evacuations and other means have diminished the excessive irritation; after which, it may again be applied, and bids equally fair for success, as in those instances in which it never disagreed.

As opium is the basis of the Thebaic tincture, it might naturally be expected that a simple solution of opium in water would produce the same good effects, but this we are told is far from being the case, such a solution having been several times applied by the author without success; mountain wine, another principal ingredient in the tincture, has likewise been tried, but without affording the leaſt relief; he therefore concludes that neither of the ingredients in the tincture are able, in a ſeparate ſtate, to produce the benefit, which they uniformly do in combination with each other, and for this reaſon he has for a long time confined himſelf to the uſe of the tincture alone. He ſuppoſes it to act at firſt by its ſtimulus, and afterwards as a ſedative, Nine caſes are related in proof of the efficacy of this remedy.

After treating of the ophthalmy, our author makes mention of the *Trichiaſis* or inverſion of the edges of the eye-lids; a diſeaſe that occaſions the hairs growing out of the ciliary edges inceſſantly to rub againſt and irritate the eye. He makes a diſtinction between an inverſion of the upper and lower lid; the former being affected by the equal, though contrary, action of the muſculus orbicularis, and levator palpebræ ſuperioris, whereas the lower eye-lid has no muſcle correſpondent to the levator of the upper. When, therefore, the trichiaſis affects the upper lid, it appears to be produced by a relaxation of the levator, and a contraction of the upper part of the orbicularis; whereas a trichiaſis of the lower lid can only ariſe from a relaxation of the ſkin, and a contraction of the lower part of the orbicularis. As theſe two caſes differ in their cauſes, the methods employed in each muſt of courſe courfe be different. In both the cure may be either palliative or radical. The former may be effected by extracting the lafhes by the root; the latter by retracting the ciliary edges, and preferving them in their natural fituation. In the trichiafis of the lower lid, the aim of the practitioner muft be to increafe the renitency of the fkin to fuch a degree as to prevent the contraction of the orbicularis; but in the trichiafis of the upper eye-lid, this would have no effect; and benefit can only be derived from adding a fufficient ftimulus to the levator palpebræ fuperioris, to excite its proper action. Of the two fpecies of trichiafis, that of the lower lid is the moft frequent. A curious cafe of trichiafis of the upper lid is related, in which, after a variety of methods had failed, a cure was effected by the following operation: an incifion was made through the integuments of the upper lid from the inner to the outer angle of the eye; the fibres of the orbicularis were then feparated fo as to denudate thofe of the levator mufcle as near to their termination in the edge of the lid as poffible; which being done, a fmall cauterizing iron, adapted to the convexity of the globe of the eye, and made pretty warm, was paffed two or three times over the tendino-carnous fibres. This

 flight

ſlight irritation produced a ſalutary contraction of the muſcle, ſo that after the inflammation ſubſided the eye became uſeful.

In a recent and ſlight caſe of trichiaſis of the lower eye-lid, our author has ſometimes accompliſhed a cure by forming a fold in the ſkin below the inverted lid, and preſerving it in that ſtate by means of ſticking-plaiſter, or of an inſtrument contrived to pinch up a ſmall portion of the ſkin, and hang on the cheek. In more ſtubborn caſes he finds it neceſſary to cut off a ſmall tranſverſe portion of ſkin below the edge of the lid, and afterwards to confine the edges of the wound together by means of a ſuture; in others of ſtill greater difficulty, as for inſtance, where the ciliary edges are not only inverted, but likewiſe contracted or ſhortened, relief can only be given by enlarging their circumference, either by an inciſion at the outer angle, or by a complete diviſion of the Tarſus in the middle. The latter operation, we are told, is not often requiſite.

Speaking of the pſorophthalmy, or inflammation and ulceration of the eye-lids, our author obſerves, that although often an effect of ſcrophula and other diſeaſes, yet it is moſt frequently a local complaint, occaſioned by an ulceration ceration of the ducts of the ciliary glands, and of courſe to be cured only by a topical application. That which he has found the moſt effectual is the *unguentum citrinum* Ph. Edin. which, when well made, forms a hard ſalve of a full yellow colour. A little of this, melted into an oil by a gentle heat, is to be rubbed upon the eye at bed-time; after which a ſoft plaiſter of the ceratum album is to be bound cloſely over the eye-lids, to prevent their adheſion to each other in the night. In the morning the eye is to be cleanſed with milk and freſh butter, well mixed together and warmed. Our author gives us ten caſes in which this practice ſucceeded.

Theſe caſes are followed by an account of the purulent eyes of new-born children. By the term *purulent*, however, Mr. Ware means only an increaſed and morbid ſecretion of mucus. In every ſtage of the diſeaſe he thinks the chief indication is to conſtringe the relaxed veſſels, and check their increaſed diſcharge. The medicine he has found to be the beſt calculated for this purpoſe is the *aqua camphorata* of Bates's Diſpenſatory, diluted according to the circumſtances of the caſe. In general he mixes a drachm of this preparation with two ounces of cold water. This compoſition is to be thrown between the eye-lids by means of a ſyringe, in ſlight caſes, once or twice a day ; in more inveterate ones, once or twice in an hour. During the ſwelling of the eye-lids he recommends, inſtead of cataplaſms, a lotion made of the curds of milk turned with alum, and an equal part of hog's lard ; this is to be applied cold, and frequently repeated, but without intermitting the uſe of the injection. Four caſes are related in which this mode of treatment was ſucceſsfully adopted.

The work cloſes with a caſe of Gutta Serena, cured by only three electric applications, each of which was continued about a quarter of an hour. The mode of electrification was firſt by carrying a ſtream of the electric fire through the eye, and afterwards by drawing ſparks from all the parts that ſurrounded it. This caſe differs from thoſe related by Mr. Hey (in the Lond. Med. Obſ. Vol. V.) in the following material circumſtances ; the diſorder came on more ſuddenly than in the caſes deſcribed by that author, the blindneſs was more entire, the eye-lids more affected, and the cure more ſpeedy.

SECTION

